# Multidrug-resistant *Salmonella* Java

**DOI:** 10.3201/eid1101.031092

**Published:** 2005-01

**Authors:** John Threlfall, Belkis Levent, Katie L. Hopkins, Elizabeth de Pinna, Linda R. Ward, Derek J. Brown

**Affiliations:** * Health Protection Agency, London, England; † Refik Saydam National Hygiene Center, Ankara, Turkey; ‡ Scottish Salmonella Reference Laboratory, Glasgow, Scotland

**Keywords:** letter, Salmonella Java, multiresistant, integrated resistance, DT104

**To the Editor**: Since 2000, *Salmonella enterica* serovar Paratyphi B variant Java (*S.* Java) with resistance to antimicrobial drugs has been isolated with increasing frequency from patients in Scotland, England, Wales, and the Channel Islands. For England, Wales, and the Channel Islands, drug-resistant *S.* Java was found in humans: 25 in 2000, 36 in 2001, 49 in 2002, and 4 in 2003 (January 1–March 31). These isolates made up 35% of 325 strains of *S.* Java in human infections over the study period (L.R. Ward, unpub. data). A range of drug-resistant spectrums (R-types) have been observed, e.g., ASSpSuTm, ASSpSuTmCp, ASSpSuTTm, ACSSpSuT (A, ampicillin; C, chloramphenicol; S, streptomycin; Sp, spectinomycin; Su, sulphonamides; T, tetracyclines; Tm, trimethoprim; Cp, ciprofloxacin) (*1*). In general, isolates of *S.* Java of R-types ASSpSuTm, ASSpSuTmCp, and ASSpSuTTm, appear to be associated with imported poultry. In contrast, infections with isolates of R-type ACSSpSuT have not been associated with poultry, and organisms with the ACSSpSuT resistant spectrum have not been isolated from poultry in the United Kingdom (R.H. Davies, pers. comm.).

In England, Wales, and the Channel Islands, *S.* Java of R-type ACSSpSuT was isolated from human patients in 64 instances from 2000 to 2003 (5 in 2000, 22 in 2001, 34 in 2002, and 3 in 2003 [to March 31]). None of these cases were related to eating contaminated foods, and the ACSSpSuT antibiogram has not been isolated from strains of this serotype from foods in the United Kingdom. This resistance pattern corresponds to that of the epidemic clone of *S.* Typhimurium definitive phage type (DT) 104 (DT 104 ACSSpSuT), which caused many infections in humans and food production animals throughout Europe, the United States, and Canada in the 1990s (*1*). In all isolates of DT104 ACSSpSuT studied, from many different countries, the resistant gene cluster has been chromosomally integrated (*1*). Resistances have been contained in a 13-kb cluster composed of 2 integrons coding for resistance to SSp (1.0 kb) and ASu (1.2 kb), with the genes for resistance to chloramphenicol and tetracyclines located between these integrons (*2*,*3*). To investigate the possibility of the horizontal transfer of the ACSSpSuT gene cluster within *S. enterica*, we have characterized the resistance genes and associated structures in strains of *S.* Java of R-type ACSSpSuT and compared them with those in a strain of DT104 ACSSpSuT

From 2000 to 2002, a total of 20 isolates of *S.* Java of R-type ACSSpSuT from patients in England and Wales (18 isolates) and Scotland (2 isolates) were characterized by phage typing, plasmid profile typing, and pulsed-field gel electrophoresis (PFGE). Pulsed-field profiles of 3 additional isolates of *S.* Java of R-type ACSSpSuT from Scotland were compared with those of isolates from England and Wales by the electronic exchange of tagged image format files (TIFFs) in a Bionumerics database. Resistance genes were identified by polymerase chain reaction (PCR) with primers specific for bla_TEM_ (A)_,_ bla_CARB-2_ (A), *cmlA* (chloramphenicol/florfenicol), *catI* (C), *catIII* (C), *aadA2* (SSp), *sul1* (Su), and *tet*G (T) (*5*). The presence of class 1 integrons was tested with the primers L1 and R1 (*2*). To identify the complete ACSSpSuT resistance gene cluster, long PCR was used on the basis of amplifying a 10,041-bp fragment of the DT104 isolate H3380 (*4*). Results were compared with those of standard strain of DT104 ACSSpSuT-P3170700, and DT104 drug-sensitive–P3343110 (*4*).

Five unrelated phage types, – 1 var 3 (6 isolates), 3b var 2 (*5*), Dundee (*2*), Worksop (*3*), and RDNC (*4*), and 3 closely-related pulsed-field types, differing by only 1 to 3 of 14 bands in the *Xba*1 PFGE profiles, were identified in the 20 isolates studied; 2 of these pulsed-field types were observed in the electronically transmitted images of the 3 isolates from Scotland. These pulsed-field profile types have been designated SPTJXB001 through SPTJXB003. Of these, SPTJXB002 predominated, being present in 11 of the isolates studied, belonging to 3 phage types. SPTJXB001 was identified in 8 isolates of 3 phage types, and SPTJXB003 in the remaining isolate. PFGE type did not change over time. All isolates were plasmid-free, and resistances were not transferable, either directly or by mobilization after a self-conjugative plasmid was introduced into the strains. By PCR, all isolates possessed bla_CARB-2_, *cmlA*, *aadA2*, *sul1*, and *tet*G but were negative for bla_TEM,_ *catI,* and *catIII*. These results corresponded to those of the control DT104 ACSSpSuT strain P3170700. When tested for class 1 integrons, all *S.* Java isolates of R-type ACSSpSuT produced characteristic amplicons of 1.0 and 1.2 kb, as did P3170700, but not the drug-sensitive strain P3343110. When tested by long PCR, all 20 *S.* Java isolates produced a 10,041-bp fragment identical to that produced by P3170700.

PCR was used to determine whether the pentaresistant phenotype was due to the presence of the *Salmonella* genomic island 1 (SGI1) as previously described (*5*). All 20 strains produced amplicons with primers U7-L12 and LJ-R1 for the left junction and primers 104-RJ and 104-D for the right junction. These results indicate that the SGI1 in the strains of *S.* Java was located in the same chromosomal location as previously described for DT 104 ACSSpSuT but lacks the retronphage found to date only in DT104 strains (*6*).

These findings demonstrated that the ACSSpSuT resistance gene cluster in *S.* Java isolated from patients in the United Kingdom from 2000 to March 2003 appeared to be chromosomally located and was almost indistinguishable from that found in the epidemic clone of DT104 ACSSpSuT. This resistance gene cluster has also been identified in strains of *S.* Agona from poultry in Belgium (*6*), in a strain of *S.* Paratyphi B from tropical fish in Singapore (*7*), and a variant cluster in a strain of *S.* Albany from fish food from Thailand (*8*). It also appears to be present in isolates of *S.* Paratyphi B of R-type ACSSpSuT from cases of human infection in France in 2003 (F. Xavier-Weill, pers. comm.). The strains of *S.* Paratyphi B from Singapore and France were not tested additionally to identify the Java variant.

The strains of *S.* Java with chromosomally integrated ACSSpSuT resistance identified in the United Kingdom are not those associated with poultry in Germany (*9*) or the Netherlands (*10*), which have also caused infections in humans in the United Kingdom (*1*). The latter strains have different phage types, resistant spectrums, pulsed-field profiles, and they possess plasmid-mediated drug-resistance. However, several *S.* Java infections in the United Kingdom have been associated with tropical fish tanks (L.R. Ward, unpub. data), although the strain has not been isolated from this medium. A substantive increase in multiresistant *S.* Java has also been reported in Australia (D. Lightfoot, pers. comm.). The antibiogram of these isolates are indistinguishable from the isolates of R-type ACSSpSuT made in the United Kingdom.

These results suggest either a common origin of the ACSSpSuT-resistance gene cluster in epidemic multiresistant DT104 and multiresistant *S.* Java or the horizontal transfer of the cluster from DT104 to other *Salmonella* serovars with a worldwide distribution. In either event, the increasing occurrence of the DT104 resistance gene cluster in potentially epidemic serovars other than *S.* Typhimurium DT104 is concerning.
